# RNF126, 168 and CUL1: The Potential Utilization of Multi-Functional E3 Ubiquitin Ligases in Genome Maintenance for Cancer Therapy

**DOI:** 10.3390/biomedicines11092527

**Published:** 2023-09-13

**Authors:** Hae Ryung Chang

**Affiliations:** Department of Life Science, Handong Global University, Pohang 37554, Republic of Korea; heyhae@handong.edu

**Keywords:** targeted cancer therapy, drug resistance, tumor suppressors, genome maintenance, cell cycle, apoptosis, ubiquitin pathway, RNF126, RNF168, CUL1

## Abstract

Ubiquitination is a post-translational modification (PTM) that is involved in proteolysis, protein–protein interaction, and signal transduction. Accumulation of mutations and genomic instability are characteristic of cancer cells, and dysfunction of the ubiquitin pathway can contribute to abnormal cell physiology. Because mutations can be critical for cells, DNA damage repair, cell cycle regulation, and apoptosis are pathways that are in close communication to maintain genomic integrity. Uncontrolled cell proliferation due to abnormal processes is a hallmark of cancer, and mutations, changes in expression levels, and other alterations of ubiquitination factors are often involved. Here, three E3 ubiquitin ligases will be reviewed in detail. RNF126, RNF168 and CUL1 are involved in DNA damage response (DDR), DNA double-strand break (DSB) repair, cell cycle regulation, and ultimately, cancer cell proliferation control. Their involvement in multiple cellular pathways makes them an attractive candidate for cancer-targeting therapy. Functional studies of these E3 ligases have increased over the years, and their significance in cancer is well reported. There are continuous efforts to develop drugs targeting the ubiquitin pathway for anticancer therapy, which opens up the possibility for these E3 ligases to be evaluated for their potential as a target protein for anticancer therapy.

## 1. Introduction

Maintenance of the genome is one of the highest priorities of the cell. The accumulation of mutations and increases in genome instability are some of the enabling characteristics of cancer [1]. When DNA damage is detected, it can halt the cell cycle of mitotic cells to prevent the error from being passed on to the next generation. If the damage cannot be repaired, cells then go through programmed cell death [2]. These normal events are altered in cancer, which enables cancer to proliferate even with the accumulation of DNA damage and abnormal physiology. These characteristics are described as “hallmarks of cancer” [1,3]. It is these very hallmarks that are utilized to distinguish cancer cells from normal cells and destroy them. Cell cycle regulation and DNA damage response (DDR) and repair are often targeted for treating cancer [4,5,6,7,8]. Although most factors involved in these pathways are considered maintenance genes, and therefore normally have tumor suppressive roles, inhibiting these factors increases the burden of genomic maintenance, and can lead to cancer cell death [2,8]. Many chemotherapeutic agents directly modify and damage DNA, increasing the genome instability of rapidly replicating cells. Targeted therapy often takes advantage of molecular targets involved in the aberrant physiology of cancer cells relative to normal cells [9,10,11]. Overexpressed, amplified or oncogenic mutations of proto-oncogenes are a favorable target for this purpose [10,12,13]. Another strategy is taking advantage of synthetic lethality, where inhibition of one gene is not enough to result in cell death, but an additional gene simultaneously does [14,15,16,17]. This phenomenon has opened up opportunities to research the non-oncogene addiction of cancer cells, in addition to oncogenes [18,19].

Increasing the number of treatment options for cancer patients is beneficial, as the genetic and molecular profile of each cancer patient is unique. Drug resistance is also an important issue, and for these reasons, identifying novel proteins for targeted therapy is continuously necessary. Ubiquitin pathway factors have been steadily investigated, as they are one of the major post-translational modifications (PTMs) that regulate a plethora of proteins in the cell [20,21,22,23,24]. Ubiquitination is essential for tagging proteins for proteolytic degradation, and it is also involved in cell signaling transduction as well as protein–protein interaction (PPI). The essential cellular functions that were mentioned above are regulated by ubiquitination, and dysregulation of this process often leads to diseases such as cancer.

Ubiquitin (Ub) is a protein of 76 amino acids in length, and it is covalently attached to the lysine residues of its substrate proteins [25,26]. A protein can be ubiquitinated on more than one lysine residue, and each lysine can be mono- or poly-ubiquitinated. There are seven lysine residues on ubiquitin, and poly-ubiquitination at K48 tags its substrate for degradation, whereas K63 functions as a signal for the recruitment of interacting partners. There are three steps to ubiquitinating a substrate protein [20,22,24,27]. Ubiquitination is a three-step event with three enzymes that are involved: E1 ubiquitin-activating enzyme, E2 ubiquitin-conjugating enzyme, and E3 ubiquitin ligases. Ubiquitin is first attached to the E1 activating enzyme, which is then transferred to the E2 conjugating enzyme. Finally, E3 ligase specifies the substrate protein and transfers the ubiquitin to the designated lysine residue to the substrate, or to another ubiquitin that has been previously attached, creating a poly-ubiquitin chain. There are estimated to be over 600 E3 ligases, with more than 300 validated enzymes [22,28]. Because of the vast number of E3 ligases, identifying the correct substrate may be a daunting task. Ge et al. has extensively classified factors involved in the ubiquitin pathway across cancer types, and showed patterns of hotspot mutations, loss-of-function (LoF) and/or both in several cancer types [29]. Because the E3 ligase recognizes a specific substrate under a given circumstance, it has the role of the specificity factor. Because of this, ubiquitin pathway factor profiling based on cancer types and detailed functional studies has enabled the development of several therapeutic agents to treat cancer, targeting specific ubiquitin pathway factors. A variety of factors involved in the ubiquitin pathway have been investigated for anticancer drug development, such as the E1 enzyme and deubiquitinating enzyme (DUB) [30,31,32]. There are several FDA-approved drugs that target ubiquitin-mediated proteolysis (UMP) factors [33], and some drugs targeting E3 ubiquitination ligases are involved in signaling or PPI [32]. This evidence shows that E3 ligases are suitable targets for anticancer treatment. The ubiquitin pathway is a part of a variety of cellular functions. The number of E3 ligases alone implies the vast number of substrates and types of modifications, some of which are tumorigenic, and some that are tumor-suppressive [23,34,35]. Ubiquitin pathway can be oncogenic or tumor-suppressive depending on the role the substrate protein has, and ubiquitination involved in signal transduction and other mechanisms is even more complex [34,35,36,37]. This is one reason to continue to investigate E3 ligases that are involved in signal transduction pathways, PPI. In addition, detailed evaluation of targeting E3 ligase in cancer with known mechanisms, particularly in signaling molecules in DNA damage repair, cell cycle regulation, and apoptosis, may provide valuable information for cancer treatment development. DDR and the DSB repair mechanisms of homologous recombination (HR) and non-homologous end joining (NHEJ) are tightly regulated by multiple E3 ubiquitin ligases [22,38,39,40]. The cell cycle is also regulated by ubiquitination, and some factors are involved in both pathways. E3 ligases are more numerous than E1 and E2 enzymes, and have specificity for its substrate. Many of these E3 ligases and their substrates have been defined [20,23,41,42,43], offering a positive outlook on the development of anticancer therapy.

This review will highlight the function of three E3 ubiquitin ligases, RNF168, RNF126 and CUL1, that have multiple roles in DDR, DNA double-strand break (DSB) repair, cell cycle regulation, and cell death. Although they have tumor-suppressive functions, they are upregulated in several cancers. LoF mutation or deletion of tumor suppressor genes is expected; therefore, the upregulation of genes may seem counterproductive for the survival of cancer cells. One hypothesis is that cancer cells’ survival in their abnormal state is dependent on certain pathways that may be not as hyperactive in normal cells. This idea is known as non-oncogene addiction [18,19,44,45,46]. Indeed, there are many chemotherapy and targeted therapy drugs that inhibit proteins involved in DDR and DNA damage repair pathways, which are considered tumor suppressors [8,19,47]. The strategy is to eliminate the gene the cancer cell is addicted to, despite its regular role. One strategy in treating cancers with DSB repair impairment is taking advantage of synthetic lethality [17,48,49,50]. For example, poly (ADP-ribose) polymerase (PARP) inhibitors (PARPi) are used in cancer patients with the BRCA1/2 mutation, which will be discussed in the later section. Similarly, targeting factors involved in DDR and DNA repair have been shown to have anticancer effects. Each E3 ligase discussed in this review is involved in multiple steps in genome maintenance, cell cycle regulation, and apoptosis, providing several pathways to an anticancer effect. Finally, this review will suggest potential strategies for utilizing these E3 ligases in cancer therapy, based on the current research developments.

## 2. E3 Ubiquitin Ligases in DSB Repair

**Figure 1:**
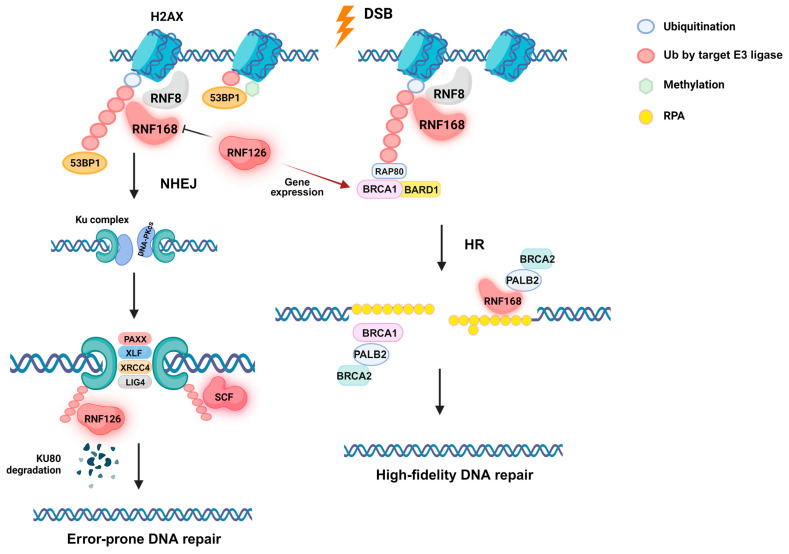
E3 ubiquitin ligase in DNA damage response and DNA double-strand break repair. Simplified depiction of E3 ligases, RNF8, RNF168, RNF126 and SCF complex (CUL1) are shown. The three main E3 ligases discussed in this review are shown in glowing red. RNF168 is recruited to RNF8-ubiquitinated γH2AX. 53BP1 can interact with the poly-ubiquitin chain attached by RNF168, or γH2AX, which is methylated and mono-ubiquitinated. The binding of 53BP1 recruits the NHEJ factor, KU 70/80 complex. The KU complex recruits DNA-PKcs, and then downstream factors. RNF126 and the SCF complex have been reported to ubiquitinate KU80 for ubiquitin-mediated proteolysis. Degradation of KU80 ensures the disassembly of the KU complex and repair of the DSB. 53BP1 binding to the poly-ubiquitinated γH2AX competes with RAP80, which recruits BRCA1-BARD1. This recruits HR factors for end resection, and exposes single-stranded DNA (ssDNA) ends. RPA binds and protects the ssDNA ends, and the BRCA1-PALB2-BRCA2 complex is recruited, bringing RAD51 to replace RPA. Downstream events allow for high-fidelity repair using homologous DNA strands. RNF126 functions in-between RNF8 and RNF168, inhibiting the recruitment of 53BP1. It is also involved in the transcription activation of BRCA1. (Created with Biorender.com accessed on 3 August 2023).

When damage occurs on the DNA, specific factors must recognize the type of DNA alteration and recruit downstream factors for repair. Ubiquitination pathway plays a key role in chromatin modification as well as protein recruitment. HR and NHEJ are two DSB repair mechanisms, which involve multiple E3 ubiquitin ligases [51]. In DDR, DSB is first recognized by the MRE11-RAD50-NBS1 (MRN) complex, which recruits the phosphoinositide 3-kinase (PI3K) family ataxia telangiectasia mutated (ATM) [52]. ATM phosphorylates the histone protein H2AX, which then recruits the mediator of DNA damage checkpoint protein 1 (MDC1) and RNF8 [38,53]. RNF8 is an E3 ubiquitin ligase, which ubiquitinates γH2AX, the phosphorylated form of H2AX [38,39,54]. Another E3 ligase, RNF168, is recruited to the mono-ubiquitinated γH2AX, and ubiquitinates it on K13-15 [55,56,57]. The schematic of E3 ligases involved in DSB repair is depicted in Figure 1. The RNF168 forms a K63 ubiquitin chain, and the poly-ubiquitinated γH2AX is recognized by downstream factors that determine the specific DSB repair pathway: HR or NHEJ. 53BP1 is recruited to the ubiquitinated H2AX, which inhibits the end resection of DNA and allows NHEJ to repair the damaged DNA [52,58,59]. It is an error-prone repair mechanism mainly functional in the G1 phase [59,60]. NHEJ does not require a homologous strand of DNA, but simply ligates the two ends of broken DNA, often resulting in the deletion of one or more nucleotides. This may result in critical mutations such as frameshifting, and CRISPR-Cas9 technology takes advantage of this phenomenon for knock-out generation [61,62]. Some cancer cells that are HR-deficient rely on NHEJ for DSB repair, and this may contribute to acceleration of genomic instability [8]. The interaction of BRCA1/BARD1 via recruitment of RAP80 [60,63,64,65] results in homologous recombination (HR), which is the major DSB repair mechanism during the S phase. which has a higher accuracy in DNA repair. HR occurs mainly in the S phase, when DNA replication occurs, and some of these factors are also involved in replication fork repair [59,60,66,67,68]. BRCA1/BARD1 functions as an E3 ligase, although its ubiquitination activity is not well studied. It has been reported that it is involved in CtIP recruitment via the MRN complex [22,63,69,70]. BRCA1-PALB2-BRCA2 is essential for RAD51 loading on to the ssDNA, and LoF results in the inhibition of RAD51 foci at DSB. Zong et al. have shown that RNF168 can act redundantly in the absence of BRCA1 by directly interacting with PALB2 to load RAD51 (Figure 1) [71]. This function will be further discussed in the designated section below.

## 3. E3 Ligases as Novel Targets

### 3.1. RNF168 and BRCA1

RNF168 is a Really Interesting New Gene (RING) class E3 ubiquitin ligase, well-established for its function in DDR. It is recruited to the mono-ubiquitinated γH2AX by RNF8, and further forms a poly-ubiquitin chain at K13-15 [55,56,57]. Both RNF8 and RNF168 share UBC13 as their E2 ubiquitin-conjugating enzyme, which contributes to substrate interaction [22,72]. It ubiquitinates histone deacetylase 6 (HDAC6) for proteolysis, which restores its interaction with H2AX [73]. RNF168 has another role downstream of DDR in DSB repair by substituting for BRCA1 in BRCA1 and 53BP1 null cells [71,74]. High RNF168 expression levels have been observed in breast cancer and esophageal cancer [75,76], as well as genetic alterations in human papilloma virus (HPV)-positive head and neck cancer [77]. It is also commonly discussed concerning PARPi resistance [71,78]. 

The DDR downstream factors 53BP1 and BRCA1 are critical for determining the mechanism of DSB repair [52,58,60,79]. It is critical that DSB is repaired by HR during the S phase when DNA replication takes place, as NHEJ is error-prone. Although HR and NHEJ predominantly function in different phases of the cell cycle, it has been experimentally shown that 53BP1 and BRCA1compete in binding to the DSB to determine the repair pathway [60,80]. 

PARP inhibition is synthetically lethal in an HR-deficient condition; for this reason, PARPi is used to treat cancers with BRCA1/2 mutations [17,49,81,82,83]. Occasionally, patients develop PARPi resistance due to an additional mutation on the BRCA1 or BRCA2 gene that later on restores its function, or epigenetic alterations that restore HR [50,84,85]. In mouse experiments, it was identified that when 53BP1 is deleted, cancer cells are re-sensitized to PARPi, and their resistance mechanism has been investigated thoroughly [17,71,80,86]. Several studies have focused on the role of RNF168 in PARPi resistance, which plays multiple roles in DDR and DSB repair [71,87]. Because NHEJ is an error-prone DNA repair system, PARP inhibition in HR-defective cells increases the accumulation mutation. Inhibition of 53BP1 blocks the path to NHEJ, and in BRCA1-deficient cells, deletion of 53BP1 revives HR [86]. It was identified that RNF168 can recruit PALB2-BRCA2-RAD51 to the DSB site in the absence of BRCA1 by directly interacting with PALB2 [71,74]. The multi-functional role of RNF168 in DDR and then during HR opens up the possibility of developing an anticancer strategy in an early stage and downstream of DSB and repair, as well as prevent drug resistance. 

Genes involved in DDR recognition and DSB repair are “caretaker” genes, and LoF mutations or deletions often contribute to tumorigenesis [80,88]. On the other hand, tumor suppressor genes or non-oncogenes are sometimes upregulated in cancer. One hypothesis for this phenomenon is that because of genomic instability and cell cycle dysregulation, cancer cells are often dependent on non-oncogenes for their survival [19]. Experimentally, it has been shown that targeting RNF168 has promising anticancer effects. RNF168 inhibition can affect DDR and thus the recruitment of both 53BP1 and BRCA1 to the DSB site. Additionally, this may be a significant therapeutic strategy for cancer patients with BRCA1 mutations, especially for those who develop PARPi resistance [78]. Targeting RNF168 ensures that even in the case that HR is restored, possibly through 53BP1 deletion or other gene alteration, it is able to inhibit both upstream at the DDR level and downstream at RAD51 loading.

The usage of a targeted therapy drug is context-dependent, and RNF168 inhibition may not be beneficial in all cancers with BRCA1 mutations. Kraise et al. have shown that overexpression of RNF168 has an anti-tumor effect on breast cancer cell lines that are BRCA1-null [87]. Although this result initially seems to contradict Zong et al.’s results, when analyzing the molecular context, the two studies display the detailed molecular context of the cancer cell. Zong et al. showed that BRCA1, lacking a RING domain, which is important for its ubiquitin ligase function, can still interact with RNF168, revealing that exon 11 is the critical region for RNF168 binding and PALB2 interaction, and not the RING domain [71,89]. Because the DSB repair mechanism is complex, with some bypass mechanism, detailed analysis of patients’ genetic and molecular profiles would be necessary for selecting RNF168 as a target for therapy.

### 3.2. RNF126

RNF126 is another RING class E3 ligase involved in DSB repair and several cellular pathways. One of the functions of RNF126 is interaction with BAG6, ubiquitinating hydrophobic substrates for proteasomal degradation [90]. It is also involved in epidermal growth factor receptor (EGFR) sorting and maintenance [91]. p21, a downstream factor of p53 and a major regulator of the cell cycle, is ubiquitinated and degraded by RNF126 [92,93]. Its upregulation and overexpression has been observed in several cancers such as bladder cancer [94], colorectal cancer [95], lung cancer [96], liver cancer [97], and poor prognosis in ovarian cancer [98]. The oncogenic function was studied in tongue cancer, suggesting the possibility of targeting RNF126 for anticancer treatment [99]. 

The involvement of RNF126 in DDR is somewhat complex. It inhibits RNF168 from H2AX ubiquitination at K13-15, consequently decreasing 53BP1 foci at the DSB site (Figure 1) [100,101]. It was also shown that knockdown of RNF126 decreased NHEJ, indicating negative regulation of DSB repair [100]. This may seem contrary to the previous claims that RNF126 could be a potential target protein for cancer therapy. Detailed inspection of the process as well as the endpoint result of RNF126 inhibition may support the potential possibility of targeting RNF126 for anticancer therapy. In NHEJ, binding of 53BP1 allows the KU70/80 complex (Ku complex) to bind to the DSB site, preventing end resection assisted by BRCA1/BARD1 and CtIP [16,23,69,102,103]. The Ku complex then recruits DNA-PKcs, then PAXX, XLF, XRCC4, and LIG4 to join the double-strand break. For NHEJ to proceed, is essential that the Ku complex is removed after recruitment of these factors, so that the DSB end is exposed for ligation [51,59,103,104]. RNF126 ubiquitinates and promotes the degradation of the KU80, which results in the dissociation of the complex. RNF126 is required for the release of the Ku complex and completion of NHEJ [40]. Lee et al. reported inhibition of NHEJ upon knockdown of RNF126 [100]. This is most likely due to the fact that removal of the Ku complex was impaired. These results show that inhibiting RNF126 may be a possible therapeutic strategy for cancer treatment. 

Studies have shown that inhibition of RNF126 suppresses cancer cell proliferation [92], and the elucidation of its function has provided researchers with details for developing a potential treatment strategy. BRCA1 mutation is an important factor of defective HR, and PARPi accelerates the accumulation of DNA damage. Degradation of RNF126 can increase PARPi sensitivity in triple-negative breast cancer (TNBC) cells [105]. RNF126 promotes transcription of BRCA1 by interacting with the transcription factor E2F1 [106]. Under this scenario, it may seem counter-productive to inhibit RNF126, as BRCA1 deficiency is one of the notable markers in breast cancer. However, understanding the relationship between PARP1 and RNF126 may clarify this conflicting results. RNF126 is ubiquitinated and degraded upon PARylation of PARP1 [105]. In cell cycle control, phosphorylation of CHK1 activates ATR/CHK1 signaling and arrests the cell cycle at G2-M. Knockdown of RNF126 decreases p-CHK1 and bypasses the G2-M checkpoint, which allows cells to re-enter mitosis. Chemotherapy drugs such as cisplatin induce DNA damage to increase the genomic instability of cancer cells. Several targeted therapy drugs inhibit DDR and DNA damage repair machinery to increase the genomic instability burden of cancer cells, leading to their death. PARPi used in BRCA1-deficient cells follows this concept. In the case of RNF126, treatment of PARPi would inhibit RNF126 degradation, allowing ATR/CHK1 activation to properly monitor the state of the cell. Depletion of RNF126 can sensitize cells to PARPi by inhibiting the ATR/CHK1 pathway, which will bypass the G2-M checkpoint and allow cells to proliferate [105]. Continuous proliferation of cancer cells under DNA-damaging drugs will further increase the genomic instability burden and potentially result in cancer cell death.

### 3.3. CUL1 and SCF Complex

**Figure 2:**
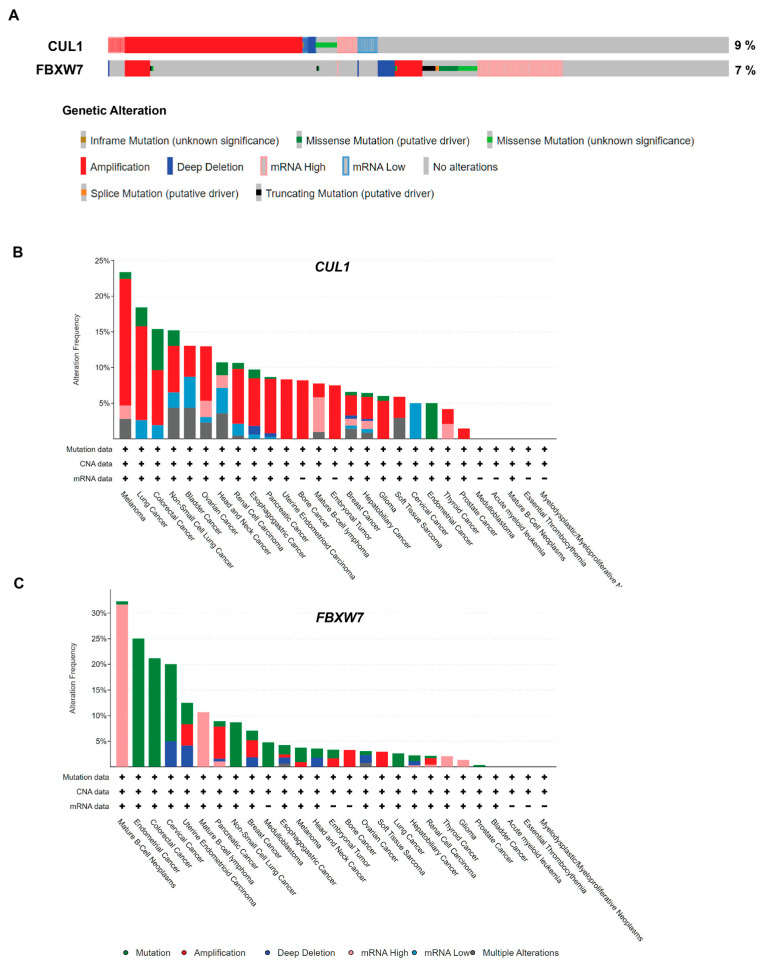
Genetic alteration of CUL1 and FBXW7 in various cancers. (**A**). Genetic alteration of SCF complex factors from the ICGC/TCGA 2020 pan-cancer study [110]. CUL1 was altered in 9% of all cases, and FBXW7 was altered in 7%. OncoPrint was generated using cBioPortal [108,109], and the total length of patient cases depicted is reduced for fit. (**B**). Percentage of CUL1 alteration across various cancer types. (**C**). Percentage of FBXW7 alteration across various cancer types.

CUL1 is part of the E3 ligase complex SCF, which includes the SKP1-CUL1-F-box, and Rbx1, which is the catalytic component [107]. There are several F-box proteins, which determine the substrate that is to be ubiquitinated [42]. CUL1 functions as a scaffold, and SKP1 is an adapter that links the two factors. An essential publication by Han Liang’s group and The Cancer Genome Atlas Research Network titled “Integrated Genomic Analysis of the Ubiquitin Pathway across Cancer Types” identified that the CUL1 hotspot mutation was enriched, and several F-box proteins were also enriched in LoF mutation and/or hotspot mutations [29]. A pan-cancer analysis by the ICGC/TCGA 2020 using cBioPortal [108,109] shows that CUL1 is altered in 9% of the samples, the majority of these being amplification and mRNA upregulation (Figure 2A) [110]. Melanoma (17.76%), lung cancer (13.16%), endometrioid carcinoma (8.33%), bone cancer (8.2%), colorectal cancer (7.69%), and pancreatic cancer (7.61%) were among the cancers that had relatively high DNA CUL1 DNA amplification (Figure 2B). For the cancer types with mRNA data, mature B-cell lymphoma showed 4.85% upregulation. Interestingly, FBXW7 mRNA was upregulated in 10.68% in the same cancer type, and 31.68% in mature B-cell neoplasms, showing upregulation of the SCF complex in B-cell related cancers (Figure 2C). Overexpression of CUL1 has been associated with poor prognosis in several cancers such as gastric cancer, breast cancer, and colon cancer, and has potential to be used as a prognosis marker [111,112,113]. Its increase was also observed in melanoma, and siRNA treatment on melanoma cell lines showed decreased cell growth, indicating its potential as a target for anticancer therapy [114]. SCF E3 ligase is a cell cycle regulator, where the F-box protein determines its substrate and ultimately which cell cycle phase it is involved in [107,115]. The F-box protein FBXW7/CDC4 is involved in Cyclin E degradation, and its LoF and/or deletion leads to premature entry into the S phase [23] and chromosomal instability [116,117].

In a colorectal cancer mouse model study, Grim et al. demonstrated that Fbw7−/− p53−/− double knock-out mice induce intestinal cancer displaying chromosomal instability. The authors stated that p53 can suppress the genomic instability that results from Fbw7 knockout. In another mouse model study, mutant Cyclin ET393A, which is unaffected by FBXW7, increased the proliferation rate of immortalized mouse embryonic fibroblasts (MEF). In p21-null MEFs, Cyclin ET393A was more stable, resulting in early entry and delayed progression of the S phase. Cyclin ET393A *p53*−/− MEFs displayed an increased cell proliferation rate, which is in line with the previous result, as p21 is downstream of p53 [118]. These two studies demonstrated the significant role of SCF E3 ligase in cell cycle control as well as genome maintenance. Fbw7 deletion itself does not cause tumorigenesis in the mouse gut, but co-deletion of Fbw7 and p53 resulted in highly penetrant and aggressive metastatic adenocarcinoma in mice [117]. 

Chromosome instability was observed in mouse model studies with *fbxw7* mutation, but direct evidence for SCF complex’s involvement in genome maintenance was later identified. RNF126’s role in KU80 ubiquitination and proteolysis has been discussed previously. It has been shown that the SCF complex is also responsible for ubiquitination of Ku80. *Xenopus laevis* extract was used to identify which F-box proteins comprise the SCF complex interacting with Ku80. Multiple F-box proteins were screened, and Fbxl12 ubiquitinated Ku80 upon DSB [119]. This interaction has not been demonstrated in the human cell context, and whether SCF^FBXL12^ ubiquitinates KU80 for proteolysis is yet to be confirmed in cancer cells. Nevertheless, SCF E3 ligase plays a significant role in both cell cycle control and genomic maintenance, making it a very attractive therapeutic agent for anticancer treatment.

## 4. Utilizing Multi-Functional E3 Ligases for Cancer Therapy

The ubiquitin pathway has been investigated for drug development for a variety of diseases including cancer [23,24,34,120]. This may come as a challenge, since an E3 ligase has multiple substrates. Developing an inhibitor that directly targets the E3 ligase may seem desirable, but in the case of cancer therapy, it may be beneficial to also consider inhibiting the downstream pathways or PPI relevant to the specific cancer’s context. Because genetic variation as well as dysregulation of cell physiology is highly dependent on each cancer patient, there may be several aspects to utilizing multi-functional E3 ligases for developing cancer therapeutics such as drugs for targeted therapy or even biomarkers for prognostic purposes and companion diagnostic markers. This section discusses ubiquitin pathway-targeting drugs and some of the strategies for the development as well as the potential utilization of RNF126, RNF168 and the CUL1 and SCF complex and its related factors for cancer therapy and diagnostics.

There are three approaches that can be taken to developing compounds that target the ubiquitination pathway: (1) inhibiting proteasome-mediated proteolysis, which prevents degradation of proteins; (2) targeted protein degradation (TPD); and (3) inhibition of the factors, especially E3 ligases. Although all three utilize the ubiquitination pathway, each takes advantage of a different aspect of this process. The drugs discussed in this section are described in Table 1. The first mechanism inhibits the proteasome, whereas the second aims to degrade a protein of interest (POI). Bortezomib [121,122,123], carfilzomib [124,125] and ixazomib [126,127] are FDA-approved proteasome inhibitors used to treat myeloma [21,32,128]. TPD can be achieved by proteolysis targeting chimera (PROTAC), a method designed to bring an E3 ligase close to the POI for ubiquitination then degradation. PROTAC consists of a E3 ligase-specific ligand and a POI ligand joined by a linker [129,130,131,132,133,134,135,136]. Arv-110, Arv-471 and CC-90009 are drugs that utilize the E3 ligase cereblon (CRBN) to degrade specific substrates [137,138,139,140,141,142,143,144]. The third approach includes inhibiting enzymatic function, blocking PPI or other mechanisms, which might be the desired mechanism for cancer therapy when considering the role of RNF126, RNF168, and CUL1.

The CRBN-targeting drugs thalidomide, lenalidomide and pomalidomide interestingly fall into the second and third approach. In TPD, PROTAC has two modalities: one is as previously described, an E3 ligase ligand–linker–POI ligand molecule; second is the “molecular glue”, which are degrader molecules used to identify the E3 ligase responsible for POI degradation [145]. Thalidomide was accidentally identified as a molecular glue, targeting CRBN in the E3 ligase complex composed of DNA binding protein 1 (DDB1), Cullin 4 (CUL4), and CRBN [146]. The PROTAC aspect of thalidomide, lenalidomide and pomalidomide results in selective degradation of POIs such as Ikaros (IKZF1), but it also inhibits autoubiquitination of CRBN [147,148]. These drugs are FDA-approved for multiple myeloma [24,149,150]. The molecular glue approach was used to identify JP-2-196, a molecule functioning as a ligand, or “handle”, for RNF126 [151]. The purpose of this study was to identify target proteins for degradation by RNF126 and molecular glue with the handle JP-2-196 by observing the phenotypic result of TPD. This may not be the appropriate approach for the specific functions of RNF126, as described previously, as inhibiting RNF126 is desirable, but not necessarily degradation of the substrate. If the Ku complex is the POI, it is possible to inhibit NHEJ in cancers defective in HR. This proof-of-concept molecule may be a promising first step, similar to CRBN molecular glues. Similar to thalidomide targeting CRBN, JP-2-196 could potentially be modified to inhibit the other functions of RNF126 discussed earlier.

Inhibiting PPI between the E3 ligase and its substrate is part of the third approach. Some examples are mouse double minute 2 (MDM2) or the human homolog HDM2, and ubiquitination of P53, one of the most prominent tumor suppressors, for proteolysis. JNJ-26854165 (serdemetan) is an MDM2 inhibitor which has undergone a phase I clinical trial [152,153,154]. Nutlin family compounds are also compounds that inhibit MDM2 interaction with p53 [120]. Drugs like BI907828 (brigimadlin), AMG-232 (navtemadlin), and HDM201 (siremadlin) are undergoing clinical trials [33,120,155,156,157,158,159,160,161,162,163,164,165]. 

RNF168, RNF126, and CUL1 are involved in several cellular functions which are critical for the hallmarks of cancer. DDR and DSB repair are essential for genome instability and mutation, evading growth suppression involves cell cycle regulators, and defective apoptosis signaling contributes to resisting cell death [1,3]. Targeting mechanisms that contribute to these hallmarks, especially inhibiting upregulated genes, is a strategy often used in targeted therapy. RNF168 is involved in recognition and marking the sites of DNA damage by recruiting downstream factors 53BP1 or BRCA1, as well as its repair via HR in substituting BRCA1 in PALB2-BRCA2-RAD51 recruitment. Considering DNA damage results in pausing the cell cycle, and can even eventually induce apoptosis if unrepairable, targeting RNF168 is one possible method to increase the genomic instability burden of cancer cells and eventually cause death. The RNF168 inhibition method may also be beneficial for patients with BRCA1 mutations who develop PARPi resistance, as its inhibition allows for BRCA1 haploinsufficiency. Targeting RNF168 in a 53BP1-null context can ensure both mechanisms of DSB repair are impaired. 

Dual regulation of both HR and NHEJ may be an effective strategy to increase the genomic instability of cancer cells, which eventually leads to death. RNF126 is involved in both HR by regulating RNF168, and NHEJ by ubiquitinating KU80 [100,101]. Its inhibition can ensure that both mechanisms of DSB repair are hindered. In addition, RNF126’s role in BRCA1 expression can cause additional impairment [106]. Inhibiting RNF126 may induce a BRCA1 downregulation environment, possibly allowing PARPi to be used as cocktail therapy. Targeting RNF126 in TNBC and improving ATR inhibitor sensitivity in breast cancer has been experimentally demonstrated, thereby potentiating its efficacy [105,166].

There are no reports on the development of small molecules that target RNF168 to date. The molecular structures of its RING domain and UDM1 domain have been determined, as well as the structures of several interacting partners, providing the foundation for structure-based drug design [72,167,168]. OTUB1 is a DUB that inhibits ubiquitination by RNF168 on γH2AX after RNF8 [169]. The inhibition mechanism is achieved by its interaction with UBC13, the E2 ubiquitin-conjugating enzyme. This interaction can be utilized for developing an RNF168 inhibitor by investigating OTUB1-UBC13 interaction, or the RNF168–UBC13 interaction interface inhibitor. Small molecules that mimic substrates or bind at available pockets have a high probably of binding to the target protein. The types of molecules that can be used to inhibit RNF168 can include small molecules, peptides or even aptamers. mRNA inhibitors such as miRNA and siRNA are also being developed for therapeutic uses, and can be considered to inhibit *RNF168* expression [170,171]. Although not a direct inhibitor of RNF168, the 1,2,3-triazole derivative “5a” tethers SQSTM1/P62 to RNF168, which induces autophagy and may serve as a potential anticancer therapeutic agent [172]. It would be beneficial to continue exploring different methods for inhibiting RNF168.

**Table 1:** Description of drugs targeting the ubiquitination pathway.

	Target	Drug	Description	Status (Phase)	Reference
Proteasome inhibitors	26S proteasome	Bortezomib	Myeloma and multiple myeloma	FDA approved	[121,122,123]
	26S proteasome	Carfilzomib	Multiple myeloma	FDA approved	[124,125]
	20S proteasome	Ixazomib	Multiple myeloma	FDA approved	[126,127]
PROTAC related	CRBN	Arv-110		NCT03888612 (1, 2)	[139,141]
		ARV-471		NCT05501769 (1), NCT05654623 (3)	[140,142,143]
		CC-90009		NCT04336982 (1, 2)	[138,144]
		Thalidomide	Multiple myeloma	FDA approved	[146]
		Lenalidomide	Refractory multiple myeloma	FDA approved	[147,148,150]
		Pomalidomide	Refractory multiple myeloma	FDA approved	[147,149]
PPI inhibitors	MDM2	JNJ-26854165 (serdemetan)	Inhibit interaction with p53	NCT00676910 (1)	[152,153,154]
		Nutlin	Inhibit interaction with p53	Preclinical	[120]
		BI907828 (brigimadlin)	Inhibit interaction with p53	NCT05613036 (1), NCT05512377 (2), NCT05218499 (2, 3)	[157,158,159,160]
		AMG-232 (navtemadlin)	Inhibit interaction with p53	NCT03217266 (1), NCT03787602 (1, 2), NCT04113616 (1, 2), NCT05027867 (2)	[155,157,161,162,163,164]
		HDM201 (siremadlin)	Inhibit interaction with p53	NCT05180695 (1, 2)	[157,165]
	FBXW7	SCF-I2	Inhibits SCF complex (Cdc4/FBXW7)	Preclinical	[173]

**Figure 3:**
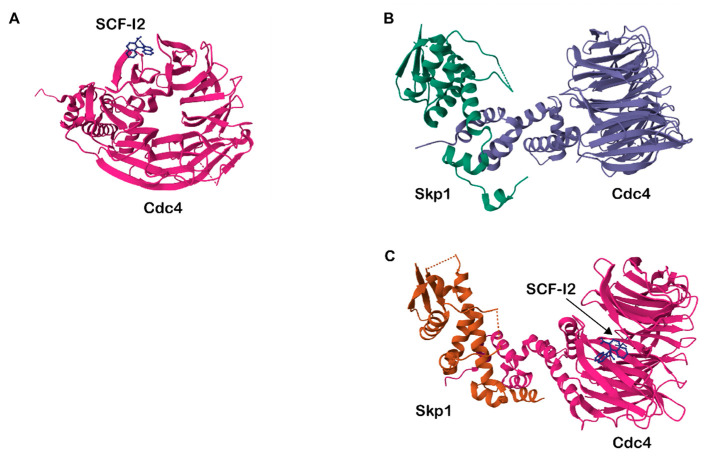
Crystal structure of Cdc/FBXW7 and inhibitor SCF-I2 (PDB ID: 3MKS). (**A**). SCF-I2 is bound to Cdc4. (**B**,**C**). Skp1-Cdc4 without and with SCF-I2, respectively. (Figure generated using RCSB PDB Mol* Viewer (WebGL version) [175,176].

The involvement of the SCF complex in multiple cellular pathways such as cell cycle control and NHEJ also makes it a considerable target. Structural analysis and compound library screening have identified a bi-planar dicarboxylic acid compound, SCF-I2, which inhibits the yeast F-box protein Cdc4 (FBXW7 in humans). The molecular structure of SCF-I2 bound to Skp1-Cdc4 has been determined by x-ray crystallography (3MKS), revealing that unlike the P53-MDM2 inhibitor, nutlin that binds at the protein–protein interaction interface, SCF-I2 inhibits Cdc4 through an allosteric mechanism [174]. A structural shift of Skp1-Cdc4 with or without SCF-I2 can be observed (Figure 3). Additionally, Pressete et al. identified a compound named PQM-277 that was predicted to bind to the CUL1-RBX1 complex. Although this compound has not been experimentally tested for its inhibitory effects, molecular docking shows promising results [173]. CUL1 has also been investigated as a prognosis marker, particularly in breast cancer and colorectal cancer [112,113]. Knockdown of *CUL1* in multiple studies has demonstrated decreased cell proliferation [111,112,113,114]. This evidence demonstrates that the SCF complex, especially that of CUL1 and FBXW7, is worthy of continuous investigation for the development of cancer-targeting therapy.

## 5. Summary

Since the identification of the mustard gas as the first anticancer chemotherapeutic drug in the 20th century, a plethora of options have been developed to treat cancer [177]. The first chemotherapy drug was found with the accidental discovery of mustard gas, which was used as a chemical weapon during World War I. Many chemotherapeutic agents have been developed since then, many of which are cytotoxic. They can be categorized into alkylating agents, antimetabolites, cytoskeleton inhibitors, and more [178]. The reason for using such cytotoxic molecules for treatment is that cancer cells rapidly proliferate. These cells require more energy, metabolize at a higher rate, and undergo much more cytoskeletal rearrangement due to continuous cell division and DNA replication. Alkylating agents modify DNA, resulting in mutations, rapid proliferation, an impaired DNA damage repair system and dysregulated cell cycle control bypassing DNA replication errors. Accelerating this process drives cancer cells to death. In a review article, Anchit Khanna describes the DNA damage threshold in human cancer cells, and discusses the transition of tumor-suppressive roles to the potentially oncogenic functioning of DDR and repair proteins, depending on the progression of cancer [179]. Studies show that therapy-related causes of secondary cancer in children due to DNA-damaging therapies such as radiation and chemotherapeutic agents are significant [180]. This highlights the need for treatment methods that minimize damage to healthy cells. The next generation of cancer therapies, such as targeted therapy and immune therapy, are continuously being developed for this reason. Targeting the genome maintenance pathway functions in a similar manner to DNA-damaging agents like platinum-based drugs and alkylating agents, in that it accelerates the accumulation of mutations [2,47]. There are many DDR inhibitors that are in use or being developed. Drugs that inhibit DDR and repair factors PARP, CHK1, ATM, ATR, and DNA-PK are some examples [2,5,8]. Synthetic lethality between DDR and repair, the cell cycle, and apoptosis in cancers can be achieved by targeting proteins that are involved in several of these pathways [15]. The three E3 ligases discussed in this review, RNF168, RNF126 and the SCF complex, particularly CUL1, are involved in recognition of DNA double-strand damage and repair, as well as cell cycle control and apoptosis. 

Like many medical interventions, drug toxicity, resistance, and side effects remain important issues in cancer treatment. Specific genetic and molecular profiles can also limit therapeutic options for patients [29,47]. Fortunately, increases in cancer genome data and translational research elucidating drugs’ mechanisms of action based on pharmacogenetics have allowed researchers to overcome this hurdle. The ubiquitination pathway is very broad, involving multiple factors, and its implications for cancer are extremely complex [35,128]. Depending on the factor and the pathway it is involved in, it can be oncogenic or tumor-suppressive [23,34]. Therefore, it is essential to specify the factors and their related pathways, as well as its interaction with substrates and the downstream effect, in order for it to be utilized in anticancer therapy. 

To increase the burden on genomic stability, administering a cocktail of drugs such as DNA-damaging agents or adjuvant therapies may enhance treatment effects. However, determining the correct drug for the complex genotype of each cancer may be difficult. One possibility is combination therapy with DNA-damaging radiation therapy and targeted therapy for DDR and DSB repair [181,182]. This idea has been explored by Fouad et al. who discussed the response of CUL1 and other cullin ring ubiquitin ligases (CRLs) after radiation therapy [183]. In TNBC, depletion of RNF126 increased sensitivity to irradiation, providing evidence for a possible treatment prospect for a type of cancer that is difficult to treat [184]. In fact, DDR and DSB repair pathway-targeting drugs can re-sensitize cancer cells to radiotherapy, and the efficacy of combination therapy with radiotherapy and other modes of treatment is actively being studied [181,185,186].

The ubiquitin pathway continues to be a sought-out mechanism used to investigate for developing anticancer therapy. It is involved in cellular functions that are often dysregulated in cancer cells, which are part of the hallmarks of cancer. Mechanisms such as DDR and DSB repair are essential for genome maintenance, and their aberration results in genomic instability. Altered expression levels of factors that are responsible for communicating between genome maintenance, cell cycle control, and apoptosis are all intricately regulated by these E3 ligases. Because E3 ubiquitin ligases provide specificity to this process, ongoing studies of these factors are necessary. Additionally, the E3 ligases that play multiple roles in mechanisms that are often utilized in cancer therapy will hopefully provide effective treatment options for difficult-to-treat cancers.

## Data Availability

Not applicable.
